# A novel molecular classification method for osteosarcoma based on tumor cell differentiation trajectories

**DOI:** 10.1038/s41413-022-00233-w

**Published:** 2023-01-02

**Authors:** Hao Zhang, Ting Wang, Haiyi Gong, Runyi Jiang, Wang Zhou, Haitao Sun, Runzhi Huang, Yao Wang, Zhipeng Wu, Wei Xu, Zhenxi Li, Quan Huang, Xiaopan Cai, Zaijun Lin, Jinbo Hu, Qi Jia, Chen Ye, Haifeng Wei, Jianru Xiao

**Affiliations:** 1 Department of Orthopedic Oncology, Shanghai Changzheng Hospital, Naval Military Medical University, Shanghai, 200003 China; 2 Musculoskeletal Laboratory, Institute of Biotechnology, University of Shanghai for Science and Technology, Shanghai, 200093 China; 3 Department of Orthopedics, Naval Medical Center of CPLA, Naval Medical University, Shanghai, 200052 China; 4 Peking-Tsinghua Center for Life Sciences, Tsinghua University, Beijing, 100084 China; 5 Division of Spine, Department of Orthopedics, Tongji Hospital Affiliated to Tongji University School of Medicine, Shanghai, 200065 China

**Keywords:** Bone cancer, Bone cancer

## Abstract

Subclassification of tumors based on molecular features may facilitate therapeutic choice and increase the response rate of cancer patients. However, the highly complex cell origin involved in osteosarcoma (OS) limits the utility of traditional bulk RNA sequencing for OS subclassification. Single-cell RNA sequencing (scRNA-seq) holds great promise for identifying cell heterogeneity. However, this technique has rarely been used in the study of tumor subclassification. By analyzing scRNA-seq data for six conventional OS and nine cancellous bone (CB) samples, we identified 29 clusters in OS and CB samples and discovered three differentiation trajectories from the cancer stem cell (CSC)-like subset, which allowed us to classify OS samples into three groups. The classification model was further examined using the TARGET dataset. Each subgroup of OS had different prognoses and possible drug sensitivities, and OS cells in the three differentiation branches showed distinct interactions with other clusters in the OS microenvironment. In addition, we verified the classification model through IHC staining in 138 OS samples, revealing a worse prognosis for Group B patients. Furthermore, we describe the novel transcriptional program of CSCs and highlight the activation of EZH2 in CSCs of OS. These findings provide a novel subclassification method based on scRNA-seq and shed new light on the molecular features of CSCs in OS and may serve as valuable references for precision treatment for and therapeutic development in OS.

## Introduction

Osteosarcoma (OS) is the most common primary bone malignancy, with an annual incidence of ~4.8 per million worldwide.^1^ Although implementation of adjuvant chemotherapy has remarkably improved OS prognosis from ~20% to over 60% in those without metastasis,^2^ chemotherapy resistance causes relapse and/or metastasis in more than 30% of OS patients, with an 5-year overall survival rate <25%.^3^ Recently, several targeted drugs, such as apatinib, sorafenib, and regorafenib, have been found to be beneficial for some OS patients, but toxic effects lead to dose reductions or interruptions in large proportions of these patients.^4–6^ Histopathologically, conventional OS is classified into three types: osteoblastic (76%–80%), chondroblastic (10%–13%) and fibroblastic (10%).^7^ However, no significant difference between the histological patterns with regard to treatment or prognosis has been observed.^7^ Subclassification of tumors based on molecular features facilitates therapeutic choice and increases the response rate in patients.^8^ Researchers have attempted to classify OS into two subtypes based on traditional bulk RNA sequencing to enhance prognostic prediction and identify relevant therapeutic targets.^9^ However, the classification specificity was relatively poor, probably due to the highly intratumoral heterogeneity and complex cell origination of OS, hindering progress in the research of the clinical significance of this subtyping method. Indeed, no classification system is currently available to guide OS treatment.

OS is considered to arise from bone marrow mesenchymal stem cell (BMSC)-derived osteoblast precursors.^10^ Studies have compared functions and gene expression between OS cells and BMSCs or osteoblasts,^11,12^ but the mechanism underlying differentiation from BMSCs or OB precursors to OS cells remains poorly understood. Disruption of normal cell fate and aberrant adoption of stem cell signals control the formation of initial cancer stem cells (CSCs), which are known to maintain self-renewal and derive other tumor cells.^13^ In addition, CSCs participate in critical steps in tumor development and progression, including chemotherapy resistance, tumor relapse, and metastatic spread.^14^ Some recent studies have reported several techniques for isolating CSCs and have described some molecular features of CSCs in OS.^15,16^ However, these isolation methods are likely to enrich the CSC sample through experimentally induced selection rather than to identify truly quiescent CSCs.^17^ Overall, a study of CSCs without preselection may promote exploration of CSC features and help in better understanding the CSC formation and differentiation into terminal cells involved in OS.

Single-cell RNA sequencing (scRNA-seq) has recently shown promise in intratumoral heterogeneity and cell differentiation trajectory exploration.^18,19^ In this study, we analyzed scRNA-seq data for osteoblastic OS and normal cancellous bone (CB) to examine a possible subclassification system based on the heterogeneity of OS cells and describe the features of CSCs in OS. According to the differentiation subsets of OS cells, we constructed a biomarker classification model for clinical prognostic prediction and treatment guidance for OS patients and further verified it in the TARGET dataset. In addition, we identified a possible CSC subset, and we describe the novel transcriptional program of this new CSC subset in OS. We hope that this new classification model and knowledge about OS stem cells described herein may serve as unique references for precision treatment in OS and for the development of OS therapy.

## Results

### Single-cell transcriptomic profiles of normal CB and conventional OS tissues

**Fig. 1:**
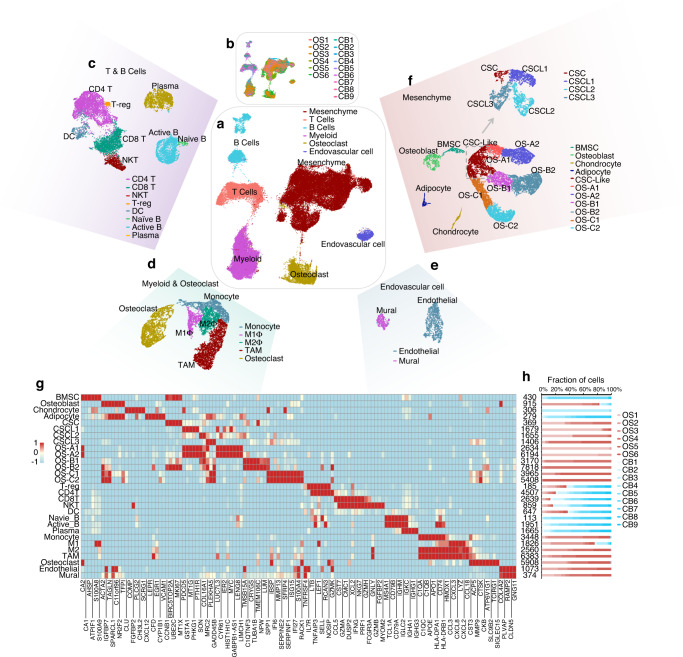
Single-cell atlas of osteosarcoma (OS) and normal cancellous bone (CB) samples. **a** UMAP plot of OS and CB transcriptomes, color-coded for six phenotypes identified by graph-based clustering. **b** UMAP plot color-coded for each OS and CB sample. **c** UMAP plot of T and B cells, color-coded for eight phenotypes identified by graph-based clustering. **d** UMAP plot of myeloid cells, including osteoclasts color-coded for five phenotypes. **e** UMAP plot of endovascular cells color-coded for two phenotypes. **f** UMAP plot of mesenchymal cells color-coded for 11 phenotypes and the UMAP plot of CSC-like clusters color-coded for 4 phenotypes. **g** Heatmap of expression of subset-specific markers across cell subsets. **h** Reproducible cell subset distributions across samples. Fractions of cells in each cluster derived from OS (red) or CB (blue) samples are shown

Normal CB tissues were obtained from nine surgical patients with degenerative disc disease. Altogether, 12 458 cells identified in the CB samples were subjected to further analysis. Single-cell transcriptomes of six conventional OS samples were obtained from the GEO dataset GSE152048.^20^ After quality filtering, we obtained 45 238 cells for subsequent analysis. The cells were classified into six distinct cell lineages annotated with canonical marker gene expression. As a result, mesenchymal, T, B, myeloid, osteoclast, and endovascular cells were identified (Fig. 1a and Fig. S1a). The distribution of the cells from each sample in the uniform manifold approximation and projection (UMAP) is shown in Fig. 1b and Fig. S1b. Compared with cells in CB tissues, mesenchymal and myeloid cells were increased and T and B cells decreased in OS samples (Fig. S1c, d).

Clustering analysis was further performed for each cell type. T cells were divided into five clusters: regulatory T cells (T-regs), CD4^+^ T cells, CD8^+^ T cells, natural killer T (NKT) cells, and dendritic cells (DCs). B cells were mainly composed of three different subgroups: naïve B cells, active B cells, and plasma cells (Fig. 1c). Myeloid cells, including osteoclasts, fell into five subsets: monocytes, M1 macrophages (M1Φs), M2Φs, tumor-associated macrophages (TAMs), and osteoclasts (Fig. 1d). Two clusters (endothelial and mural cells) were identified among endovascular cells (Fig. 1e). Mesenchymal cells were divided into BMSCs, osteoblasts, adipocytes, chondroblasts, and seven OS tumor cell clusters, which we named CSC-like, OS-A1, OS-A2, OS-B1, OS-B2, OS-C1, and OS-C2. The CSC-like clusters were further subdivided into four subclusters (CSC, CSCL1, CSCL2, and CSCL3) based on clustering (Fig. 1f). Thus, 29 clusters were revealed in CB and OS tissues.

Each cluster exhibited a distinct gene expression pattern (Fig. 1g and Table S1). Correlation analysis showed that clusters from the same cell lineage had higher similarity than those from other cell lineages (Fig. S1e), confirming the reliability of the clusters. The distribution of unique molecular identifiers in each cell lineage is shown in Fig. S1f. Each cluster included cells from multiple patients, showing clear distribution differences between OS and CB samples (Fig. 1h). The proportion of all cell clusters in each sample is also provided in Fig. S1d.

### The distinct transcriptome program in mesenchymal cells

**Fig. 2:**
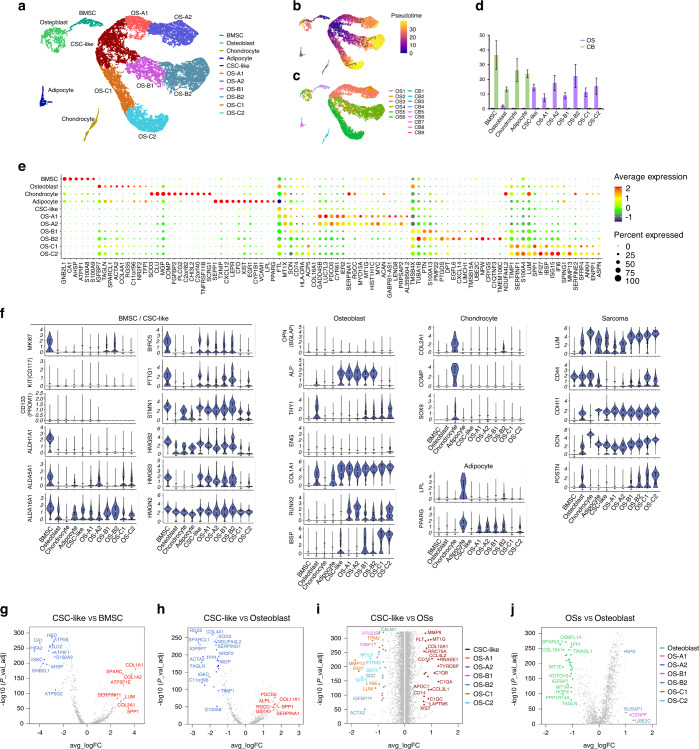
Differences in cell composition and gene expression between OS and CB samples. **a** UMAP plot of mesenchymal cells by the Monocle3 method. **b** Pseudotime trajectory of mesenchymal cells. **c** UMAP plot of mesenchymal cells color-coded for each sample. **d** Differences in cell proportion between OS and CB samples in each mesenchymal cluster. **e** Dot-plot heatmap of the most significant genes of each cluster in mesenchymal cells. **f** Violin plots showing differential expression of stem cell, osteoblast, chondrocyte, adipocyte, and sarcoma markers in each mesenchymal cluster. **g** Differences in gene expression between CSC-like and BMSC clusters. **h** Comparison of gene expression between CSC-like and osteoblast clusters. **i** Differences in gene expression between the CSC-like cluster and total OS cells. **j** Differences in gene expression between total OS cells and osteoblasts. *P < 0.05

Mesenchymal cells are the main constituent of OS samples (Fig. S1c), which is consistent with the mesenchymal origin of OS.^21^ Using the Monocle3 method, we observed that OS tumor cells included a CSC-like cluster and three branches with two clusters in each branch (Fig. 2a). Pseudotime trajectory analysis (monocle3 method) and velocity analysis both demonstrated the CSC-like cluster to be the origin of each branch during cell differentiation (Fig. 2b and Fig. S2a). We found that the clusters from each branch observed by the monocle3 method were also concentrated in different differentiation branches of the monocle2 method (Fig. S2b). Interestingly, OS cells from various samples were enriched in distinguished branches (Fig. 2c), indicating the heterogeneity of the OS samples. Cell proportion analysis demonstrated the OS cell clusters mainly derived from OS samples and the normal mesenchymal clusters from CB samples (Fig. 2d).

The most significant markers of each mesenchymal cluster are shown in Fig. 2e and Table S2, demonstrating clear distinctions between the clusters. Further analyses showed the stem cell markers to be highly expressed in BMSCs and CSC-like clusters and partly expressed in OS-A1/A2/B1/B2 clusters (Fig. 2f), suggesting that the OS cells in branch-A/B seemed to have stronger stemness than those in branch-C. In addition, chondrocyte and adipocyte markers were expressed at low levels in all OS cell clusters; OB markers ALP, THY1, and COL1A1 were expressed in OS cell clusters (Fig. 2f), suggesting the osteoblastic nature of OS cells.^22^ We observed that ALP and COL1A1 were upregulated in OS cells compared with OBs, indicating enhanced extracellular matrix formation ability in OS cells, which might induce abnormal osteogenesis in OS.^22,23^ Sarcoma markers were differentially expressed in OS cell clusters (Fig. 2f), suggesting the heterogeneity of the three branches. Some of the gene expression changes during the OS cell differentiation trajectories are shown in Fig. S2c. For example, MYC and CYR61 were upregulated during differentiation trajectory-A; CDK4 was gradually increased in branch-B, and expression of TIMP3 and MMP13 was gradually enhanced in branch-C.

We further compared gene expression between OS and normal mesenchymal cells and found that CSC-like cells expressed higher levels of the osteoblastic markers COL1A1, COL1A2, and SPARC than BMSCs (Fig. 2g and Table S3). Compared with OBs, CSC-like cells overexpressed SPP1 and SERPINA1 genes related to tumor progression (Fig. 2h and Table S4). Compared with other OS cells, CSC-like cells highly expressed chemoresistance genes, including FTL, XIST, and MT1G (Fig. 2i and Table S5). Overall, osteoblastic marker expression exhibited limited diversity between OS cells and OBs (Fig. 2j and Table S6). A comparison of gene expression between BMSCs and other normal mesenchymal cells (including adipocytes, chondroblasts, and OBs) is shown in Fig. S3a–c and Tables S7–9.

### Specific molecular features of the three differentiation branches of OS cells

**Fig. 3:**
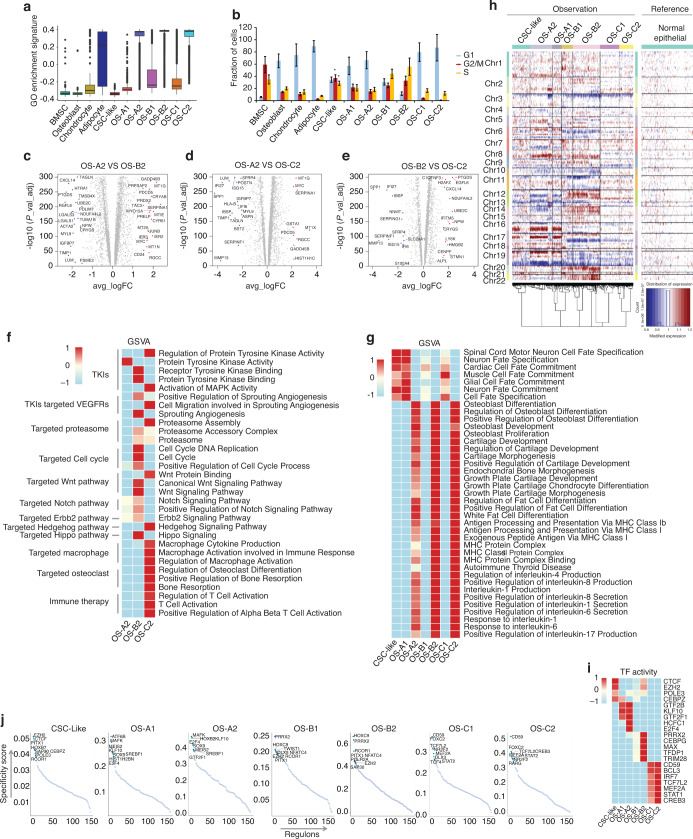
Transcriptional distinction between the three differentiation trajectories of OS cells. **a** Differences in total GO pathway enrichment between the seven OS clusters. **b** Differences in the proportion of cell cycle phases in the 11 mesenchymal clusters. **c** Differences in gene expression between the OS-A2 and OS-B2 clusters. **d** Differences in gene expression between the OS-A2 and OS-C2 clusters. **e** Differences in expression between the OS-B2 and OS-C2 clusters. **f** Heatmap showing differences in the activation of pathways related to targeted therapies in OS-A2/B2/C2 clusters calculated by the GSVA method. **g** Heatmap showing differences in activation of pathways between the seven OS clusters. **h** Heatmaps of differences in single-cell copy number between the seven OS clusters and normal endovascular cells. **i** Heatmap showing differences in TF activity between the seven OS clusters. **j** TF activity in the seven OS clusters. The top 8 activated TFs were marked in each cluster

We performed GSVA in the seven clusters of OS cells to further analyze the features of the three branches of OS cells (Fig. S2d). Total GO pathway enrichment increased gradually in the CSC-like, OS-A1/B1/C1, and OS-A2/B2/C2 clusters (Fig. 3a). Because quiescence is a critical feature of stem cells,^24^ this result suggests the primitiveness of the CSC-like cluster and OS-A1/B1/C1 clusters as the primary stage during OS cell differentiation.

We further studied each cluster’s cell cycle stage distribution and found that BMSCs and CSC-like cells were enriched in the S and G2/M stages, indicating activation of cell proliferation, which is consistent with their stem cell nature (Fig. 3b). Interestingly, we found that OS-B1/B2 cells were concentrated in the S stage but that OS-C1/C2 was mainly in the G1 stage (Fig. 3b), indicating that OS cells in branch-B might have strong proliferative activity and that the proliferation of cells in branch-C may be possibly slow. The differentially expressed genes in the OS-A2/B2/C2 clusters are illustrated in Fig. 3c–e and Tables S10–12. We observed that several bone metabolism-related genes, including SPP1, IBSP, and MMP13, were upregulated in the OS-C2 cluster compared with the other two clusters.

When focusing on specific pathway activation among the OS cell clusters, CSC-like and OS-A1 exhibited stronger pluripotency than other clusters, and pathways related to osteoblast development and bone morphogenesis were activated in OS-A2/B2/C2 clusters, possibly revealing the maturation of these clusters (Fig. 3g). In addition, we analyzed pathways related to targeted therapies in OS-A2/B2/C2 clusters and found obviously different pathway enrichment between the OS-A2/B2/C2 clusters, such as activation of cell cycle- and ERBB2-related pathways in OS-B2 and enrichment of bone resorption and immune activation pathways in OS-C2 (Fig. 3f). Expression levels of target genes involved in targeted therapies were also diverse in the OS-A2/B2/C2 clusters. For example, VEGFRs (FLT1 and KDR) and CDK4 were overexpressed in the OS-B2 cluster, and PD-L1 (CD274) was upregulated in the OS-C2 cluster (Fig. S2e). These results suggest that OS cells in distinct differentiation branches might be sensitive to different drugs.

Differences in gene expression in cells are often caused by changes in chromosome copy number variation (CNV). According to the CNV calculation results based on scRNA-data, we found that CSC-like cells had different CNVs compared with other OS clusters. In addition, the clusters in the same differentiation branch exhibited similar CNVs, but different differentiation branches had distinct CNVs. For example, extensive chromosomal gains were observed in 6p, 8q, 16q, 17p, and 19p of branch-A clusters, 3p, 6, 10, and 19 of branch-B clusters, and 3p, 9q, and 19 of branch-C clusters. Extensive chromosomal losses were found in 10, 12q, and 19q of branch-A clusters, 8q, 12p, 14, 20, and 22 of branch-B clusters, and 12q of branch-C clusters (Fig. 3h). These results highlight the heterogeneity between the three differentiation branches of tumor cells in OS.

We performed transcription factor (TF) analysis for the seven OS cell clusters to reveal the transcriptional program during OS cell differentiation. The results highlighted the relative activation of EZH2 and CTCF in CSC-like cells (Fig. 3i), which correlated with tumor cell stemness, proliferation, and drug resistance.^25,26^ Moreover, clusters in the same differentiation branch shared similar TF activation, whereas those in different branches exhibited different TF activation, demonstrating that the transcriptional programs in the three differentiation trajectories are different (Fig. 3i, j). TF activation of the four normal mesenchymal clusters (BMSCs, osteoblasts, chondroblasts, and adipocytes) is shown in Fig. S3d, e.

### OS classification based on the differentiation branches of OS cells

**Fig. 4:**
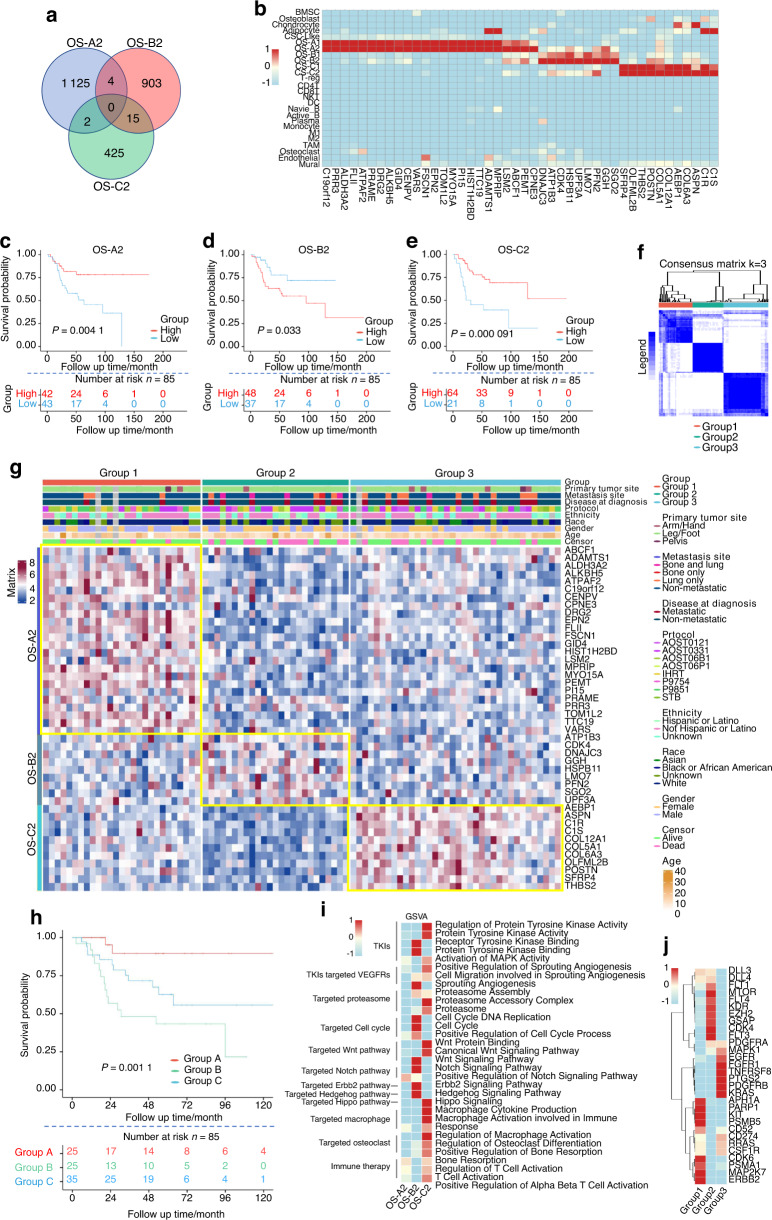
Classification of OS samples from the TARGET dataset based on the markers of the three differentiation branches of OS cells. **a** Venn diagram showing the similarities and differences of the calculated markers of OS-A2/B2/C2 clusters. **b** Heatmap showing the expression of the 44 selected marker genes of OS-A2/B2/C2 in all 29 clusters. Kaplan–Meier curves of overall survival for patients with different expression levels of total OS-A2 (**c**), OS-B2 (**d**) or OS-C2 (**e**) markers in OS patients from the TARGET dataset. **f** Consensus clustering matrices with the 44 marker genes of OS-A2/B2/C2 in TARGET OS samples for *k* = 3. **g** Heatmap showing the expression of 44 marker genes in the three clustered groups of TARGET OS samples. **h** Kaplan–Meier curves of overall survival for the three clustered groups of TARGET OS patients. **i** Heatmap showing activation differences of pathways related to targeted therapies in the three subgroups of TARGET OS samples. **j** Heatmap showing expression levels of genes related to targeted therapies in the three subgroups of TARGET OS samples

Due to the clear differences in gene expression, TF activation, and CNV between the three branches of OS cells, we considered that the OS samples could be classified into three groups based on the differentiation branches of tumor cells. The intersections between the marker genes of OS-A2/B2/C2 were quite limited (Fig. 4a), suggesting that OS-A2/B2/C2 markers can be used to identify differentiation branches in OS samples. We selected the 44 most specific markers of the OS-A2/B2/C2 clusters, including 24 markers for OS-A2, 9 for OS-B2, and 11 for OS-C2 (Fig. 4b). Interestingly, we observed that the markers of OS-B2 correlated significantly negatively with the survival of OS patients based on the TARGET dataset (Fig. 4d); conversely, the markers of OS-A2 and OS-C2 predicted good prognosis in OS patients (Fig. 4c, e).

To explore the possible application of OS-A2/B2/C2 markers in OS sample classification, we first divided 88 OS samples in the TARGET dataset into three groups by resampling selected tumor profiles using the 44 selected markers for OS-A2/B2/C2. The three subtypes achieved a good discrimination effect for OS samples (Fig. 4f), and the markers of OS-A2/B2/C2 were overexpressed significantly in Group A/B/C samples (Fig. 4g). Consistent with the relevance between the markers of OS clusters and the prognosis of OS patients, Group B patients exhibited a significantly worse survival rate than the other two groups (Fig. 4h). Pathway enrichment analysis showed that pathways related to targeted therapies were differentially activated in the three groups of OS samples, which was partly similar to the pathway activation in the OS-A2/B2/C2 clusters, such as activation of cell cycle pathways in Group B and activation of bone resorption and immune activation pathways in Group C (Fig. 4i and Table S13). The target genes of targeted therapies were also differentially expressed in the three group samples: VEGFRs (FLT1, KDR, FLT3, and FLT4) and CDK4 were overexpressed in Group B samples, and PD-L1 (CD274) was upregulated in the Group C cluster (Fig. 4j). These findings suggest that the classification system based on OS-A2/B2/C2 markers may be used to guide clinical treatment for OS patients.

### Verification of the classification system in clinical OS samples

**Fig. 5:**
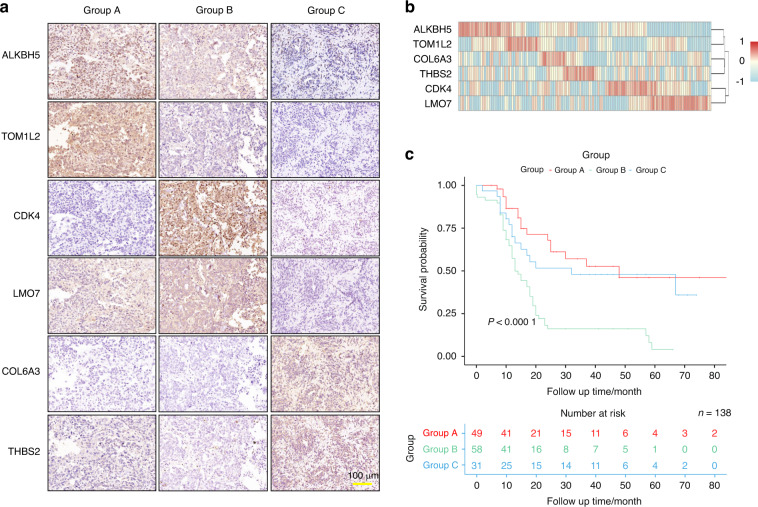
Group classification of OS samples based on IHC staining. **a** IHC staining of ALKBH5, TOM1L2, CDK4, LMO7, COL6A3, and THBS2 in three OS samples from each group (No. 13, No. 68, and No. 79 of OS samples). **b** Statistical analysis of expression of six gene markers in each OS sample. **c** Kaplan–Meier curves of overall survival for the three groups of OS patients

To further examine the clinical significance of the classification system in OS samples, we performed Immunohistochemical (IHC) staining for six gene makers selected from the OS-A2/B2/C2 clusters (OS-A2: ALKBH5 and TOM1L2; OS-B2: CDK4 and LMO7; OS-C2: COL6A3 and THBS2) in 138 osteoblastic OS samples (Fig. 5a). OS patients were divided into three groups according to the strongest expressed marker of each sample. Groups A/B/C highly expressed the markers of the OS-A2/B2/C2 clusters, which parallels Group A/B/C in the TARGET OS cohort. We observed some correlations between the expression of markers for the same cluster (Fig. 5b). By using K-M curves, we found that the OS patients in Group B exhibited significantly worse overall survival than those in the other two groups (Fig. 5c), similar to the survival analyses in the TARGET dataset (Fig. 4h). Except for alive status, no significant difference in clinical characteristics was observed among the three groups of OS patients (Table S14). These results suggest that the classification of OS samples can be performed through IHC staining, which may help in the prognostic evaluation of OS patients.

### Identification and characterization of OS stem cells

**Fig. 6:**
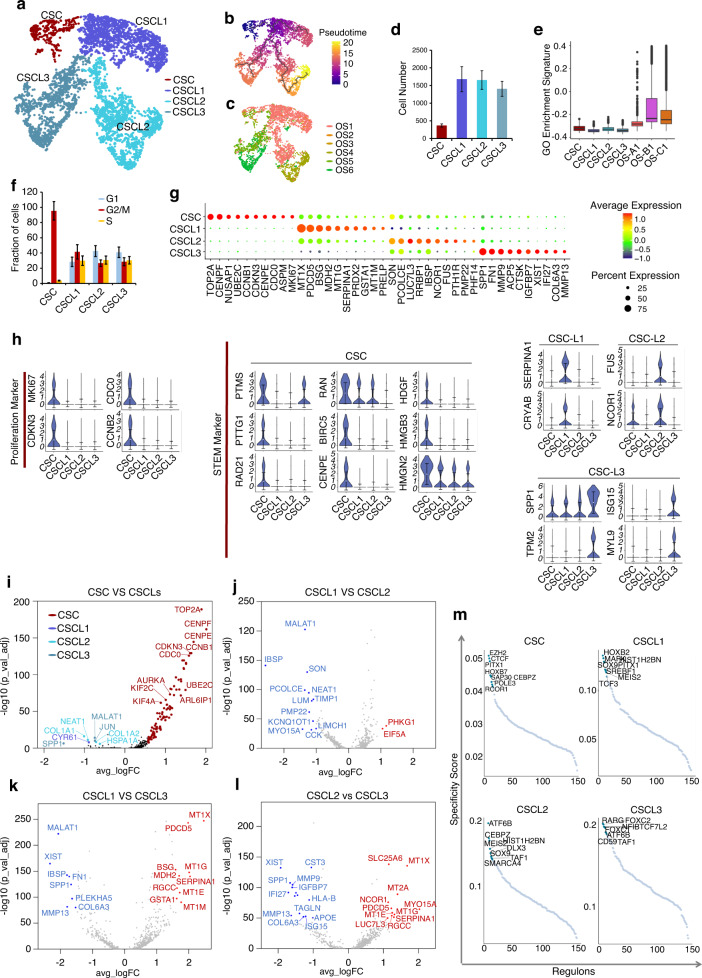
Molecular features of OS stem cell CSC-like cells. **a** UMAP plot of CSC-like cells using the Monocle3 method. Four subclusters were identified in CSC-like cells by graph-based clustering. **b** Pseudotime trajectory of CSC-like cells. **c** UMAP plot of CSC-like cells color-coded for each OS sample. **d** Mean cell numbers of the four subclusters of CSC-like cells in OS samples. **e** Total GO pathway enrichment among the CSC, CSCL1, CSCL2, CSCL3, and OS-A1/B1/C1 clusters. **f** The cell proportion of each cell cycle phase in four subclusters of CSC-like cells. **g** Violin plots showing expression of proliferative markers and stem cell markers in the four subclusters of CSC-like cells. **h** Dot-plot heatmap of the most significant genes in the four subclusters of CSC-like cells. **i** Differences in gene expression between CSCs and other CSC-like subclusters. **j** Differences in gene expression between CSCL1 and other CSCL2 clusters. **k** Differences in gene expression between CSCL1 and other CSCL3 clusters. **l** Differences in gene expression between CSCL2 and other CSCL3 clusters. **m** TF activity in the four subclusters of CSC-like cells. The top 8 activated TFs are marked in each cluster

As CSCs play a critical role in tumor development and progression,^27^ we further studied the composition of the CSC-like cluster and found that they can be further classified into four clusters: CSC, CSC-like 1 (CSCL1), CSCL2, and CSCL3 (Fig. 6a). Pseudotime trajectory analysis identified three differentiation branches of CSCs that correlated with CSCL1, CSCL2, and CSCL3 (Fig. 6b). The CSC cluster contained 369 cells, which accounted for 0.82% of all cells in OS (Fig. 6d). CSCL1 cells were mainly from samples enriched in OS-A1/A2 cells (OS1 and OS2), over 50% of CSCL2 cells were from samples enriched in OS-B1/B2 cells (OS3 and OS6), and most CSCL3 cells were from samples enriched in OS-C1/C2 cells (OS4 and OS5) (Fig. 6c). In addition, the gene markers of CSCL1/2/3 were partly similar to OS-A2/B2/C2 clusters, such as MT1G and SERPINA1 in CSCL1, as well as SPP1 and MMP13 in CSCL3 (Figs. 2e and 6h and Table S15). These results suggest that CSCL1/2/3 might be the progenitors of OS-A2/B2/C2.

Total GO pathway enrichment in four CSC-like clusters was lower than that in OS-A1/B1/C1 (Fig. 6e). Cell cycle analysis showed the CSC cluster to be arrested in G2/M phase compared with the CSCL1/2/3 clusters (Fig. 6f). As G2/M arrest has been found in some cancers and CSCs, we considered that the G2 checkpoint is a possible therapeutic target for anti-CSC therapy of OS. Furthermore, CSCs overexpressed the cell proliferation markers MKI67, CDC20, CDKN3, and CCNB1 and expressed more stemness markers than CSCL1/2/3 clusters (Fig. 6g). Differences in gene expression between the CSC and CSCL1/2/3 clusters are shown in Fig. 6i–l and Tables S16–19, demonstrating overexpression of genes related to the mitotic cell cycle in CSCs, such as TOP2A, CENPE, CENPF, and CCNB1. Pathway analysis also revealed DNA binding and chromosome condensation to be activated in the CSC cluster (Fig. S4a and Table S20). These results suggest that the CSC cluster might comprise a primary stem cell subset.

TF analysis was performed to detect specific TF activation in CSCs. It was found that TF was related to drug resistance, DNA methylation, and MSC differentiation, including EZH2, CTCF, PITX1 and HOXB7,^28–32^ which were obviously activated in CSCs compared with in CSCL1/2/3 clusters (Fig. 6m). We further detected activation and expression of EZH2, CTCF, PITX1, and HOXB7 in all mesenchymal clusters and found them to all be significantly overexpressed and activated in CSCs compared with in other mesenchymal clusters (Fig. S4b). These results describe the particular transcriptional program in CSCs.

### Distribution change and molecular features of macrophages and osteoclasts in OS

**Fig. 7:**
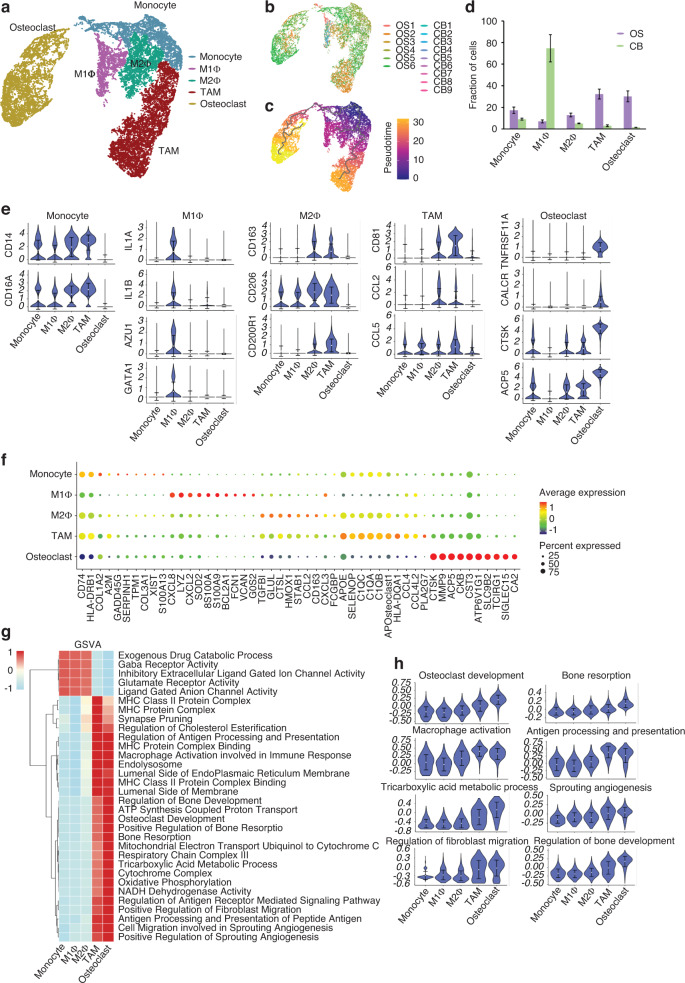
Activation of tumor-associated macrophages (TAMs) and osteoclasts (OCs) in OS. **a** UMAP plot of myeloid cells, including OCs, using the Monocle3 method. **b** UMAP plot of myeloid cells color-coded for each OS and CB sample. **c** Pseudotime trajectory of myeloid cells. **d** Differences in cell proportion between OS and CB samples in myeloid cell clusters. **e** Violin plots showing differences in expression of monocyte, M1Φ, M2Φ, TAM, and OC markers in each myeloid cluster. **f** Dot-plot heatmap of the most significant genes in the five myeloid clusters. **g** Heatmap showing differences in pathway activation between the five myeloid clusters calculated by the GSVA method. **h** Violin plots showing differences in activation of important pathways between the five myeloid clusters, including OC development, bone resorption, macrophage activation involved in immune response, regulation of antigen processing and presentation, tricarboxylic acid metabolic process, positive regulation of sprouting angiogenesis, positive regulation of fibroblast migration, and regulation of bone development pathways (part of the pathway is abbreviated in the figure)

Macrophages and osteoclasts play crucial roles in tumor immunity and tumor-bone interactions in OS.^33,34^ Five classical clusters (monocytes, M1Φs, M2Φs, TAMs, and osteoclasts) were identified in this cell lineage (Fig. 7a). Each cluster was composed of cells from multiple samples (Fig. 7b). Monocle analysis revealed two differentiation trajectories of monocytes, differentiating into osteoclasts or macrophages (Fig. 7c), which is consistent with the known differentiation directions of monocytes.^35^ Monocytes, M2Φ, TAMs, and OCs were increased and M1Φ decreased in OS compared with CB tissues (Fig. 7d), possibly promoting the malignant behavior of OS, as M1Φs function as a tumor suppressor by activating the antitumor immune response.^36^ Each cluster was characterized by a distinct gene expression pattern with known markers, such as CD14 and FCGR3A in monocytes; IL1A and IL1B in M1Φs; CD163 and MRC1 in M2Φs; CD81 and CCL2 in TAMs; and TNFRSF11A, CTSK and ACP5 in OCs (Fig. 7e, f and Table S21).

GSVA was performed to detect the function of macrophages and osteoclasts. Consistent with the known roles of each cluster, pathways of bone resorption were activated in osteoclasts and pathways of the immune response in TAMs (Fig. 7g, h and Table S22). We also found that pathways related to the regulation of angiogenesis, fibroblast migration, and bone development were activated in osteoclasts (Fig. 7g, h), suggesting that osteoclasts may play a role in promoting angiogenesis and tumor progression in OS.

### Perturbation of lymphocytes and endovascular cells in OS

Eight clusters were identified in T and B lymphocytes, including T-reg, CD4^+^ T, CD8^+^ T, NKT, DC, naïve B, active B, and plasma cells. Each cluster comprised of cells from multiple samples (Fig. S5a). The clusters were characterized by distinct gene expression (Fig. S5c and Tables S23 and 24), and the reported markers of lymphocytic clusters were verified to be expressed in specific subgroups (Fig. S5e). Nearly all T- and B-cell clusters were decreased in OS compared with in CB tissues (Fig. S5d), suggesting possible immunosuppression in OS.

Two clusters (endothelial and mural cells) were identified in endovascular cells (Fig. S5b), with both being supported by known and novel markers (Fig. S5c, e and Table S25). Possibly due to a sufficient blood supply of the tumor, both clusters of endovascular cells were significantly increased in OS compared with CB tissues (Fig. S5d).

### Ligand‒receptor mediated intercellular interactions in the OS microenvironment

**Fig. 8:**
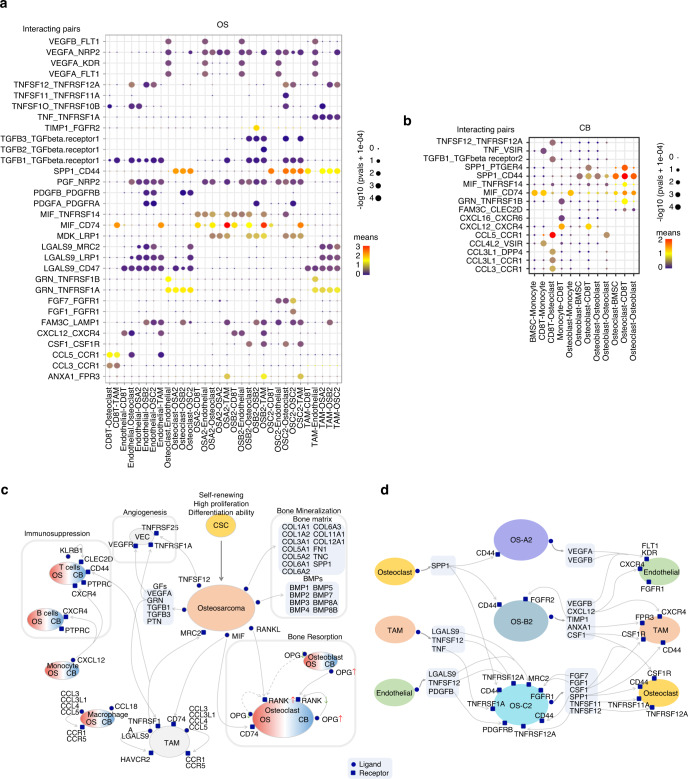
Ligand-receptor interactions between clusters in OS and CB samples. **a** Dot-plot heatmap shows part of the ligand-receptor interactions between the clusters calculated by the CellPhoneDB method. **b** Dot-plot heatmap showing part of the ligand-receptor interactions between clusters in CB samples. **c** Schematic representation of differences in the microenvironment between OS and CB samples. **d** Schematic representation of differences in intercellular interactions between OS-A2/B2/C2 and other cell clusters in OS samples

CellPhoneDB analysis was performed to detect interactions between clusters. Overall, intercellular interactions in OS samples were activated compared with those in CB samples; endothelial, osteoclast, and TAM cells exhibited relatively abundant interactions with OS cells (Fig. S6a). Part of the ligand‒receptor interactions between the clusters in OS and CB tissues are depicted in Fig. 8a, b. It was found that the tumor cells in OS samples exhibited a stronger ability to regulate angiogenesis, macrophage activation, and bone resorption than the osteoblasts in CB samples. For example, OS cells secreted VEGFA, GRN, and TNFSF11, which bind to KDR on endothelial cells, TNFRSF1A on TAMs, and TNFRSF11A on osteoclasts (Fig. 8c). However, lymphocytes were more activated in CB samples, such as the CXCL12-CXCR4 interaction between monocytes and lymphocytic subsets in CB samples (Fig. 8c), suggesting immunosuppression in OS.

In addition, communications between OS-A2/B2/C2 and other subsets were obviously different. For example, OS-A2 and OS-B2 expressed more VEGFA/B, which activate their receptors on endothelial cells and might promote angiogenesis. OS-B2 expressed CXCL12 and might activate TAMs and CD8 T cells by binding to CXCR4. OS-C2 markedly expressed TNFSF11 and CSF1, which promotes osteoclast formation through activated TNFRSF11A and CSF1R. Self-interactions of TIMP1-FGFR2 and FGF7-FGFR1 were also observed in OS-B2 and OS-C2, respectively (Fig. 8a, d). The ligand‒receptor interactions between OS-A2/B2/C2 and endothelial cells, TAMs, and osteoclasts are shown in Fig. S6b. These results suggest that the tumor microenvironment (TME) is different between the three subgroups of OS samples, which might provide clues for therapy for the three subgroups.

## Discussion

Although advances in chemotherapy have substantially improved the survival of OS patients, prognosis with chemoresistance or recurrence remains poor.^37^ Targeted therapies have shown effectiveness in some OS patients.^38,39^ However, it is difficult to predict the availability of chemotherapy or targeted therapies before treatment. A new classification of OS based on molecular mechanisms may facilitate the treatment choice and prognostic prediction for OS patients.

Some studies have attempted to classify tumors based on the transcriptome of whole samples,^40,41^ but a complex TME might confound the heterogeneous features of tumor cells, possibly limiting the clinical significance of these classification methods. The development of scRNA-seq has led to research of tumor action mechanisms. Indeed, scRNA-seq has been used to analyze the differentiation trajectory of tumor cells and cell interactions in the TME.^42,43^ Studies have shown that tumor cells from different samples have diverse differentiation trajectories with distinct molecular features,^44,45^ indicating the possible classification of tumors based on the differentiation trajectory of tumor cells. Nevertheless, there is limited research on tumor classification based on scRNA-seq. In the present study, we identified subtypes of OS samples based on the features of three differentiation branches of OS cells and analyzed potential treatment for each subtype, as that scRNA-seq can better reflect tumor cell metabolism and TME changes compared with the traditional transcriptome of samples.

In the present study, we analyzed the cellular dynamics and molecular features of six conventional OS samples, compared them with nine CB tissues by using scRNA-seq technology, and observed three distinct differentiation directions of OS cells from CSCs. OS cells from each sample were concentrated in specific differentiation branches, which suggested that OS samples can be divided into three subgroups based on the three differentiation branches of OS cells. Further analyses showed that OS patients in the three subgroups had different prognoses, pathway activation, and gene expression levels relative to the targeted and immune therapies.

Samples in Groups 1 and 3 in the TARGET cohort were characterized by the markers of OS-A2 and OS-C2, respectively. We observed that the mitotic cell cycles in these two branches were suppressed compared with branch-B and CSC-like cells but that the prognosis of Group A and C patients was better than that of Group B patients. As all six OS patients had received chemotherapy before surgery, we considered the tumor cells in these two branches to be sensitive to conventional chemotherapy, suppressing tumor cell proliferation and leading to a relatively good prognosis for the patients in these two groups.

Tumor cells in branch-A and branch-C exhibited different pathway enrichment relative to targeted and immune therapies. No relatively activated pathway of targeted therapy was observed in OS-A2 cells or Group A samples, though immune response and bone resorption pathways were activated in OS-C2 cells. Furthermore, analysis of genes related to targeted and immune therapies showed HER2 (ERBB2), KIT, and PSMA1 to be relatively overexpressed in OS-A2 and Group A samples, suggesting the possible role of therapies in targeting HER2, KIT, and proteasome in Group A patients.^46–48^ PDGFRB, FGFR1, and CD274 were also relatively overexpressed in OS-C2 and Group C samples, suggesting the possible role of therapies in targeting PDGFRB, FGFR1, and PD-L1 in Group C patients.^49–51^ Expression of genes related to therapies exhibited partial differences compared with activation of related pathways, possibly due to the complex genetic composition of the pathways. In addition, cell‒cell interaction analyses showed that OS-C2 cells obviously expressed signaling molecules that stimulate osteoclasts and macrophages, consistent with the pathway activation in OS-C2 cells and Group C samples and suggesting that therapies targeting bone resorption, such as denosumab,^52^ might be valuable for Group C OS patients.

Samples in Group B were characterized by markers of the OS-B2 cluster. Cells in the S and G2/M phases of the cell cycle were increased in OS-B1 and OS-B2 cells, indicating activation of cell proliferation. As all six OS patients received chemotherapy before surgery, we considered that the effect of chemotherapy was limited to OS-B2 cells. Consistent with the possible chemoresistance of OS-B2 cells, OS patients in Group B exhibited a significantly worse prognosis than those in the other two groups. Cell cycle pathways were relatively activated both in OS-B2 cells and Group B samples. CDK4 and EZH2 were relatively overexpressed in OS-C2 and Group B samples, suggesting the possible role of therapy targeting cell cycle markers, such as CDK4 and EZH2.^53,54^

Due to the potential benefit of this classification model of OS in clinical treatment, we further evaluated a method for distinguishing OS samples. Through IHC staining of the six markers selected from OS-A2/B2/C2 clusters in 138 OS samples, we observed that most OS samples highly expressed only one group of markers matched to OS-A2, OS-B2, or OS-C2, suggesting that the three subgroups of OS can be identified by IHC staining. We also found that Group B samples in the Changzheng Hospital cohort exhibited worse overall survival than the other two groups, consistent with the results of the TARGET dataset analysis. These results suggest that the classification model of OS might easily be applied in clinical practice.

As CSCs are considered the origin of other tumor cells,^13^ analysis of CSCs may help in deciphering the program that initiates cells to develop into a heterogeneous tumor mass. In addition, CSCs are a cluster with high proliferative ability and drug resistance and are critical in carcinogenesis, including tumor propagation, recurrence, and metastasis.^55,56^ The roles and mechanisms of some molecular markers have been studied in CSCs in OS.^57,58^ However, the complex heterogeneity of tumor cells may cause experimentally induced selection when specific molecular markers are used to identify CSCs.^17^ The development of scRNA-seq makes it possible to view heterogeneity at even deeper levels^59,60^ and provides new insight into the description of CSCs.^61,62^ Regardless, no study has reported scRNA-seq analysis of CSCs in OS.

Analysis of mesenchymal cells in OS tissues in this study revealed a CSC-like cluster with high proliferative ability, stemness, and pluripotency. In addition, we categorized the CSC-like cluster into four clusters: a CSC and three CSC-like clusters. The differentiation trajectory, gene expression, and pathway enrichment analyses all demonstrated the CSC cluster to be SCs of tumor cells in OS. Not surprisingly, most pathways related to chemotherapy and targeted therapy were not activated in CSCs, suggesting that most chemotherapies and targeted therapies may not be able to suppress the proliferation of CSCs.

We also observed that the CSC cluster exhibited a specific transcriptional program. EZH2 is the most significantly activated TF in CSCs and controls methylation of histone 3,^63^ promotes cell proliferation,^64^ and regulates osteoblast differentiation.^65^ EZH2 promotes tumor cell proliferation, invasion, and resistance to chemotherapies^66,67^ and predicts poor prognosis in OS patients.^68^ High EZH2 expression is a characteristic of CSCs in ovarian and prostate cancers.^69,70^ Due to the critical role of EZH2 in tumors, EZH2 inhibitors have been used in the treatment of advanced hematologic and solid tumors in some clinical trials.^71,72^ However, limited studies have addressed the role of EZH2 in OS stem cells. Our results identified EZH2 as the most characteristic feature of CSCs, suggesting that EZH2 inhibitors are a potential treatment option for tumor stem cells in OS. In addition to EZH2, several specific TFs, such as CTCF, PITX1, and HOXB7, play key roles in drug resistance, DNA methylation, and MSC differentiation in CSCs.^28–30,32^ These results shed new light on the transcriptional program of CSCs in OS and provide novel potential therapeutic targets for OS.

The findings of our study may provide new insight into the heterogeneity of OS samples and serve as possible guidance for OS treatment. Nevertheless, there are some limitations in this study. First, scRNA-seq data were obtained from only six conventional OS patients. More scRNA-seq results from OS samples are required to further verify and improve the classification of OS samples. In addition, the OS patients in this study received different chemotherapies, which may cause heterogeneity among the tumor cells and microenvironments of the different samples. Finally, as the pathological type of the OS samples in TARGET was not indicated, some of the TARGET samples might not be suitable for the present classification system. Further studies should be planned based on the current results. First, the effects of the possible drugs targeted to CSCs and each OS subgroup must be examined in relevant OS cells and OS mouse models, and well-designed clinical trials are needed to verify the efficacy of the potential drugs in each subtype and confirm our hypothesis.

In conclusion, we constructed a novel classification model of OS samples based on tumor cell differentiation branches, which was further verified in the TARGET dataset and OS samples. We identified three subtypes of OS and found that they exhibit distinct prognoses and different expression levels of therapy-related genes, suggesting that the new grouping system may be able to provide important prognostic information and have a certain guiding significance for the treatment of OS. Furthermore, we identified the CSC cluster in OS tissues and described its transcriptional program, which may help develop new potential therapeutic targets for tumor stem cells in OS.

## Methods

### Patient samples

For scRNA-seq, nine normal CB samples were obtained from patients with degenerative disc disease who underwent spinal surgery at Changzheng Hospital (Shanghai, China) from December 2018 to September 2021. For IHC staining, 138 OS samples were obtained from OS patients who underwent tumor resection surgery in Changzheng Hospital from January 2014 to December 2018. The patient characteristics are summarized in Table S26. Patient consent was obtained for the study, and the sample collection was under ethical approval. This study was approved by the Research Ethics Committee of Shanghai Changzheng Hospital.

### Cell isolation and scRNA sequencing

CB samples were sent to the laboratory within 1.5 h after collection, and all samples were washed with PBS three times to remove impurities. According to the standard 10x Genomics sample preparation process, the samples were cut into 1–2 mm pieces and then digested with type IV collagenase and trypsin. For complete digestion, they were incubated in a shaker at 37 °C and shaken every 10 min. After digestion and incubation for ~1–2 h, the cell suspension was filtered through a strainer and centrifuged to remove the enzymes. Red blood cell lysis buffer was added to the cell suspension to eliminate red blood cells. The number of cells in the supernatant was counted by using a Countess II Automated Cell Counter, and only samples with an appropriate cell density (1 000 cells per μL) and a live-cell percentage greater than 90% were subjected to further scRNA sequencing. Then, the single-cell gel beads-in-emulsion (GEMs) were generated after the 10X Genomics Chromium single-cell controller was used for processing with standard samples. For individual GEMs, cells were lysed, releasing RNA that was captured and barcoded by the reverse transcription process. Following Single-Cell 3′ Reagent Kit V3 User Guide, we processed the mixture and constructed libraries with Single 3′ Library and Gel Bead Kit v3. The cDNA libraries were sequenced using an Illumina Nova6000 at a sequencing depth of at least 100 000 reads per cell. Finally, CellRanger software (v3.0.2; 10X Genomics) was used to demultiplex raw data generated by sequencers into FASTQ files and quantitate the gene expression profile for each cell.

### GEO dataset

The GEO database was screened for 10x genomics sequencing data of OS, and the GSE152048 dataset was screened out. Among them, six tumor tissue samples from six patients pathologically diagnosed with conventional OS were chosen to explore the cellular composition combined with normal CB samples. The characteristics of the OS samples are summarized in Table S27.

### Quality control, batch correction, and clustering

Analysis of the scRNA-seq data was performed in the R statistical environment (v3.6.3). The raw data for 15 samples were processed separately with the Seurat method of data cleaning. To remove low-expressed genes and low-quality cells, we retained genes expressed in at least 3 cells and filtered the cells with more than 20% mitochondrial reads and less than 5% ribosomal reads. In addition, we deleted cells with fewer than 200 genes or more than 5 000 genes and doublets that were detected with DoubletFinder (https://github.com/chris-mcginnis-ucsf/DoubletFinder). Then, we used the *NormalizeData ()* function to normalize the count data with the LogNormalize method selected and the *FindVariableFeatures ()* function to screen out 1 000 variable genes utilized in principal component analysis. We passed the Seurat object consisting of the 15 data to the *RunHarmony ()* function, which is supported by Harmony (https://github.com/immuno-genomics/harmony); the “plot_convergence” parameter was set as TRUE to integrate the batch effects. *FindNeighbors ()* constructed a shared nearest neighbor graph with Harmony reduction and 50 dimensions input. The same parameters were also used in the formation of the UMAP. Classification of all cells was manually labeled according to the characteristics of expression. We used dimensionality reduction and the cell clustering method provided by Monocle3 downstream to reanalyze the tumor cells and the CSC-like subgroup distinguished from the tumor separately. Similarly, the batch effect from samples was eliminated by running the align_cds() function.

### Calculation and display of differentially expressed genes

We used the *FindAllMarkers ()* and *FindMarkers ()* functions of the scran package to perform a Wilcoxon test between pairs of cell clusters to find genes specifically expressed in each cluster. For endovascular cells and Clara cell populations subdivided by Monocle3, we mapped the grouping information of these cell subgroups back to the Seurat object and calculated differential genes for the Seurat object that rewrites the grouping information. According to the results of the calculation, the ggplot2 and heatmap packages were used to visually display heatmaps, violin plots, and bubble maps.

### Pathway enrichment

To assess gene expression signatures and pathway activation, GSVA was performed using gene sets of the C2 and C5 collections obtained from the molecular signature database to assess the activation level of the relative pathway in each cell and visualize it through a heatmap.

### Regulon activity analysis

The PySCENIC (V1.22) algorithm combined with the Arboreto package GRNBoost2 method was used, and the cis-Target human motif database (V9) was used to build the gene regulatory network (GRN) in all cells. Raw expression data and labeled clusters were extracted from the Seurat data and Monocle3 data.

We performed filtration with the default parameters of the pySCENIC pipeline. Then, we used the grnboost2 method to compute the GRNs. CisTarget databases containing hg38__refseq-r80__10kb_up_and_down_tss.mc9nr.feather and hg38__refseq-r80__10kb_up_and_down_tss.mc9nr.feather and the TF motif annotation database (v9) were used to identify enriched motifs. The Aucell function was applied to score all cells to show regulon activities. Finally, the similarity score was calculated for the regulons in each cluster and transferred to the Specific score based on Jensen–Shannon divergence.

### Analysis of the cell differentiation trajectory

We used the Monocle3 (V0.2.3.0) algorithm to order cells along the trajectories based on the pseudotime in mesenchymal cells. The expression matrix of the mesenchymal cells derived from the Seurat object was passed to Monocle3. We used the *new_cell_data_set ()* function to create a cds object and perform dimensionality reduction, cell clustering, and differentiation trajectory inference.

### Chromosome copy number variation analysis

We employed the inferCNV (V1.6.0) method with recommended parameters for 10X data to illustrate the diverse patterns of chromosome CNV in tumor cell clusters. Mural and endothelial cells were used as the reference.

### TARGET-OS dataset

We screened the OS sequencing data from Genomic Data Commons (GDC) Data Portal and screened out the TARGET OS cohort. The standardized RNA-sequence FPKM and clinical files were downloaded from GDC Data Portal on January 30, 2021. A total of 85 OS samples with complete clinical follow-up information were obtained.

### Kaplan‒Meier survival curve analysis

The downloaded TARGET OS data were normalized and then integrated. For the integrated dataset, Kaplan‒Meier survival curves of different subtype gene sets in the dataset were drawn with the survival package. The OS rate from diagnosis to death or the last follow-up was calculated.

### Immunohistochemistry

Samples were fixed with 4% paraformaldehyde, dehydrated through a graded series of ethanol, paraffin-embedded, and sliced into 5-mm sections. IHC staining for ALKBH5 (ab195377, Abcam, USA), TOM1L2 (ab121716, Abcam), CDK4 (ab199728, Abcam), LMO7 (ab224113, Abcam), COL6A3 (ab231025, Abcam) and THBS2 (ab112543, Abcam) was carried out using standard histological procedures described in the manual for the Histostain-Plus (DAB) kit (Mingrui Biotech, China). The staining degree of each protein was calculated by using ImageJ software.

## Supplementary information

Supplementary Table 27

Supplementary Figure 1

Supplementary Figure 2

Supplementary Figure 3

Supplementary Figure 4

Supplementary Figure 5

Supplementary Figure 6

Supplementary Table 1

Supplementary Table 2

Supplementary Table 3

Supplementary Table 4

Supplementary Table 5

Supplementary Table 6

Supplementary Table 7

Supplementary Table 8

Supplementary Table 9

Supplementary Table 10

Supplementary Table 11

Supplementary Table 12

Supplementary Table 13

Supplementary Table 14

Supplementary Table 15

Supplementary Table 16

Supplementary Table 17

Supplementary Table 18

Supplementary Table 19

Supplementary Table 20

Supplementary Table 21

Supplementary Table 22

Supplementary Table 23

Supplementary Table 24

Supplementary Table 25

Supplementary Table 26

Supplementary Data

## Data Availability

The osteosarcoma data are available at the NCBI Gene Expression Omnibus (GEO) database under accession ID GSE152048. The raw sequencing data for cancellous bone samples are available from National Genomics Data Center (NGDC) under accession number PRJCA011197.
