# Supplementary figures and images for: A novel molecular classification method for osteosarcoma based on tumor cell differentiation trajectories
Source: Bone Res. 2023 Jan 2;11:1. doi: 10.1038/s41413-022-00233-w (PMC9806110; doi:10.1038/s41413-022-00233-w)

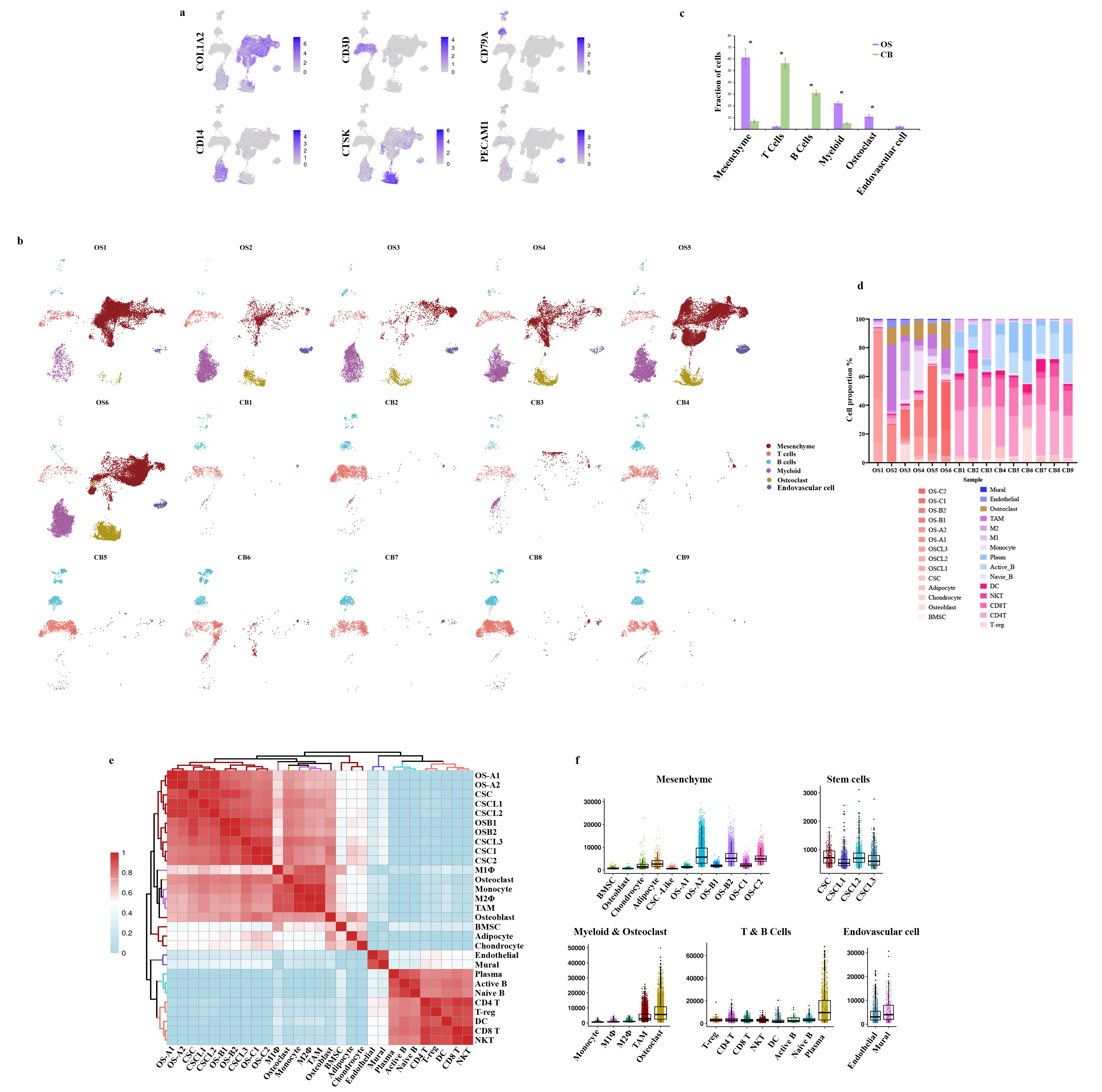

Supplement: Supplementary file 2 — Supplementary Figure 1 [file 41413_2022_233_MOESM2_ESM.jpg]

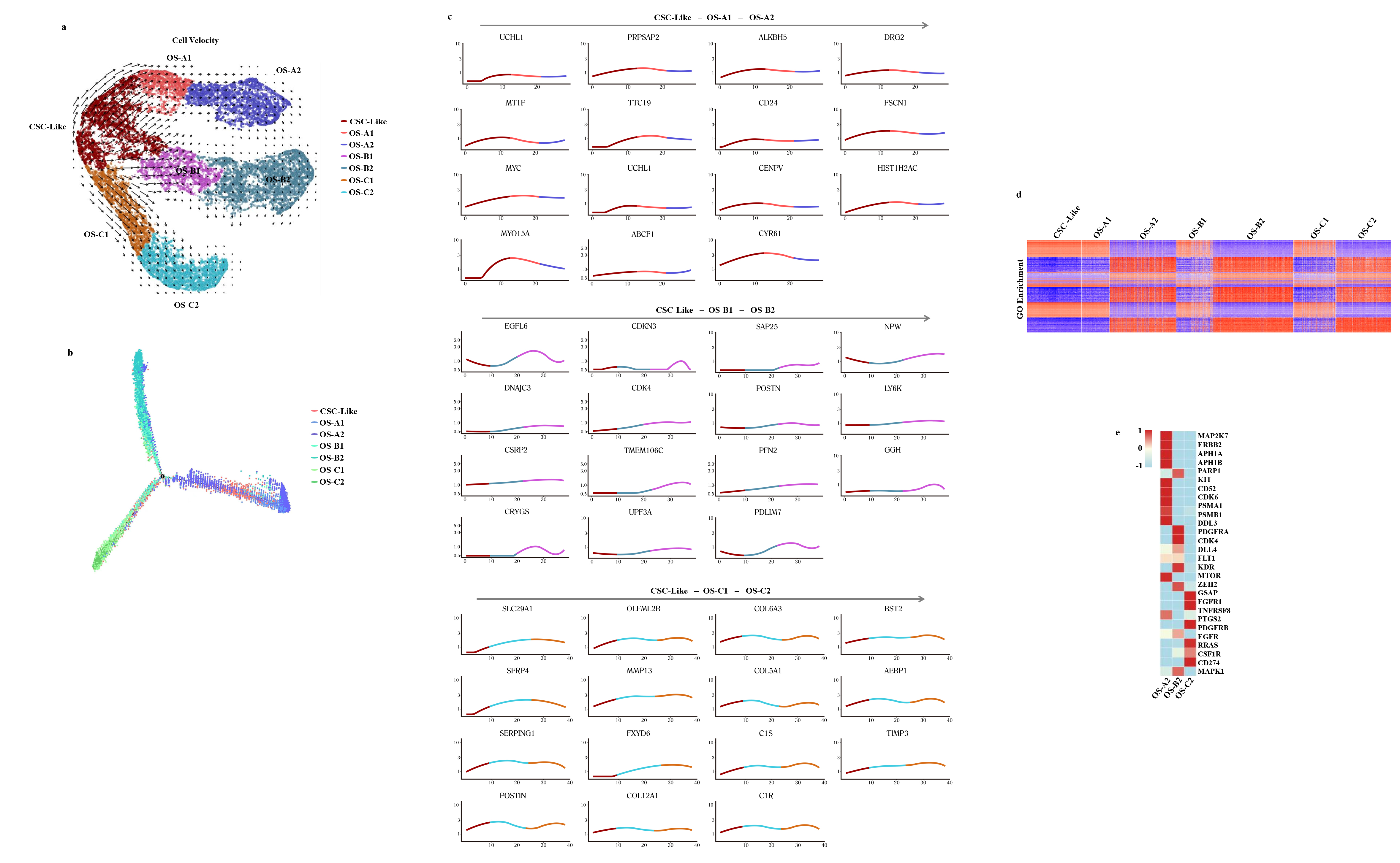

Supplement: Supplementary file 3 — Supplementary Figure 2 [file 41413_2022_233_MOESM3_ESM.jpg]

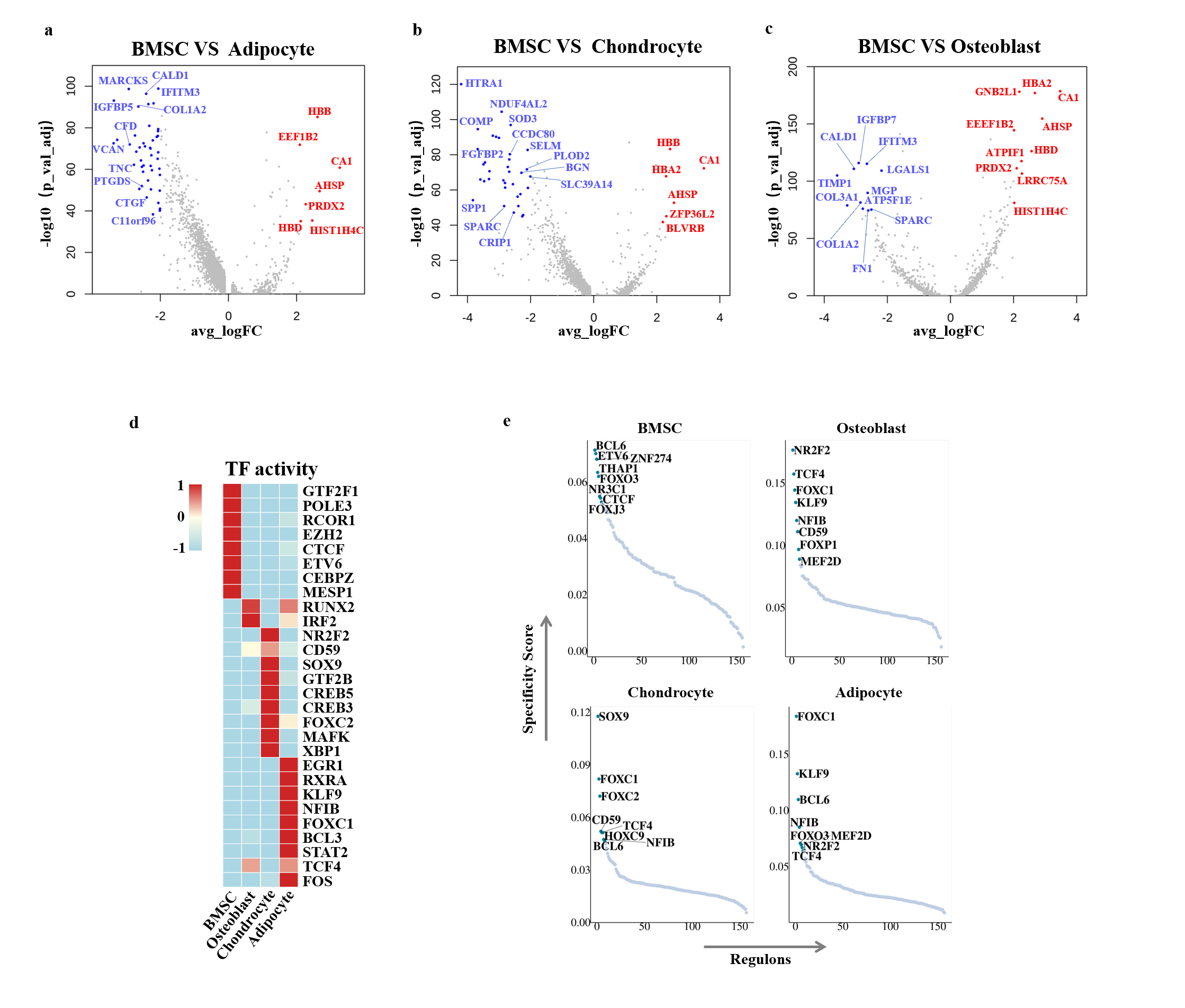

Supplement: Supplementary file 4 — Supplementary Figure 3 [file 41413_2022_233_MOESM4_ESM.jpg]

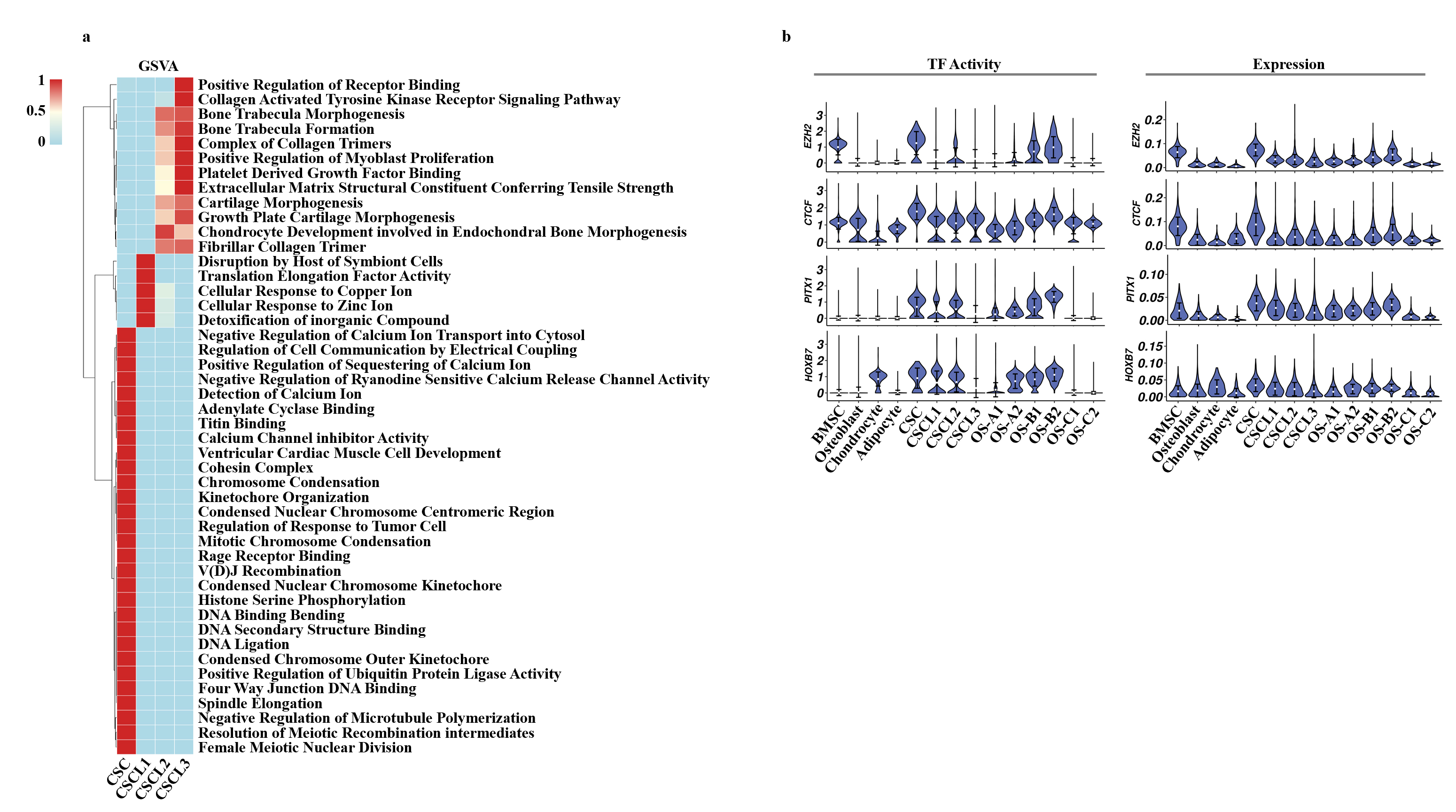

Supplement: Supplementary file 5 — Supplementary Figure 4 [file 41413_2022_233_MOESM5_ESM.jpg]

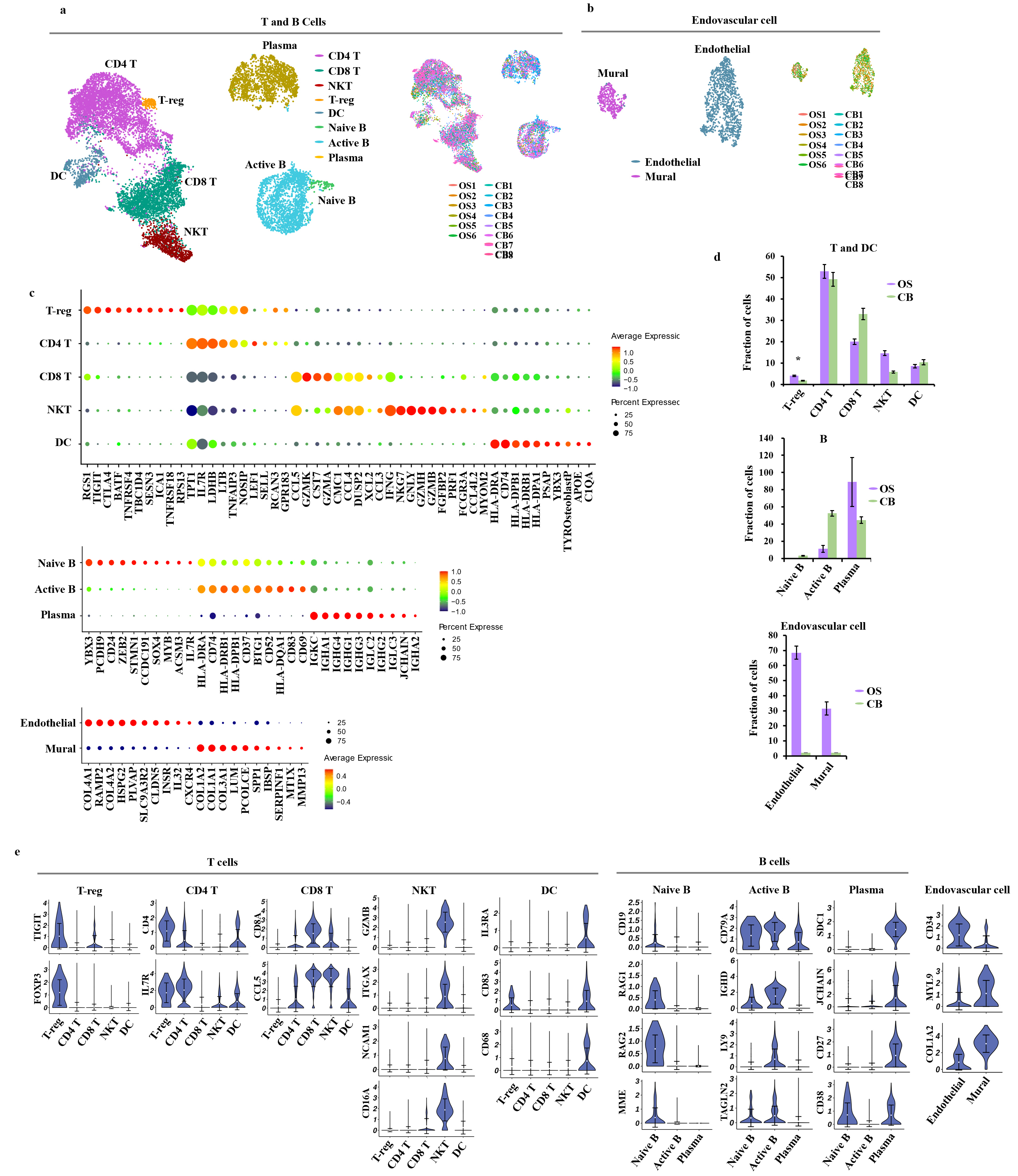

Supplement: Supplementary file 6 — Supplementary Figure 5 [file 41413_2022_233_MOESM6_ESM.jpg]

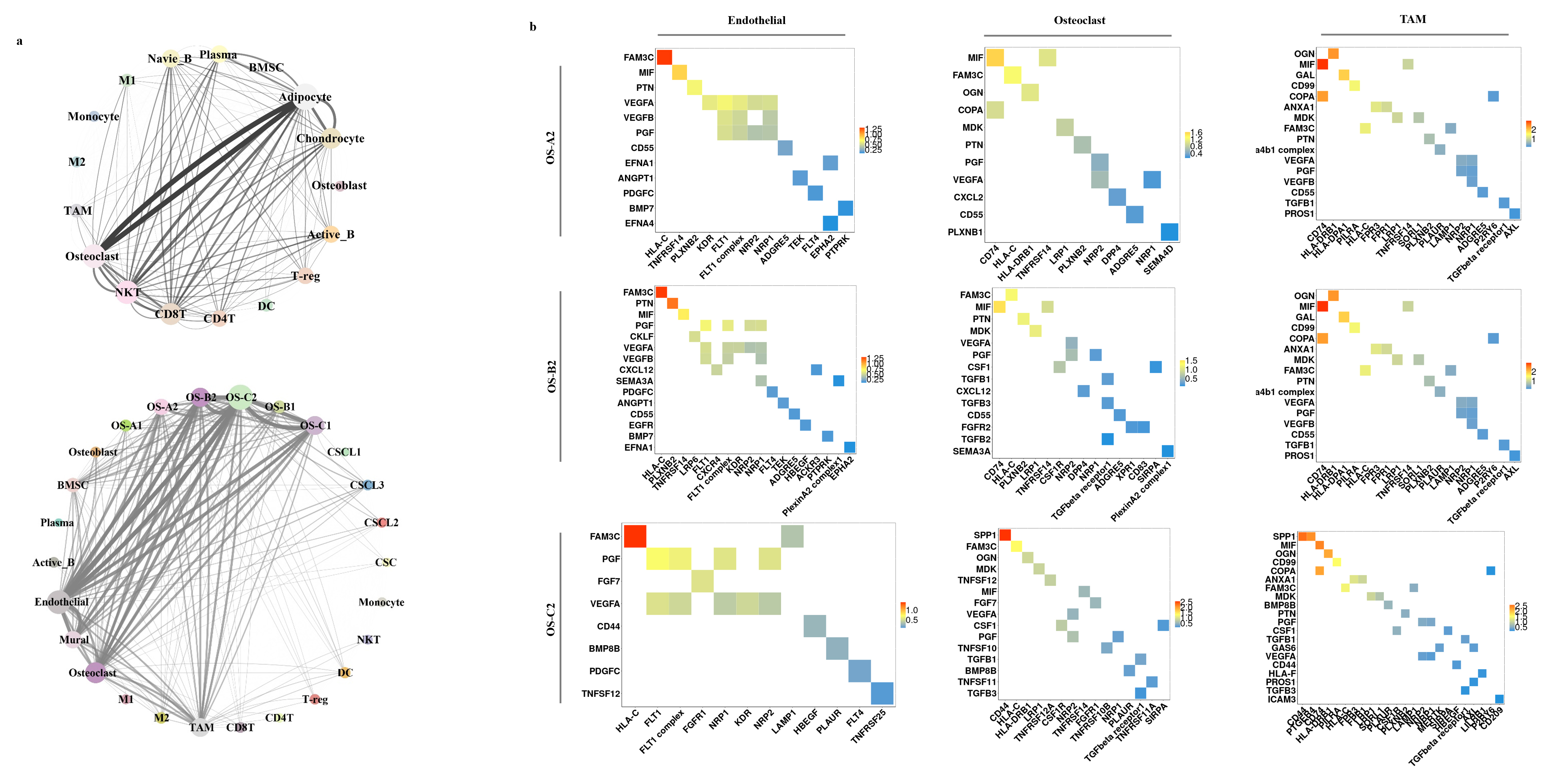

Supplement: Supplementary file 7 — Supplementary Figure 6 [file 41413_2022_233_MOESM7_ESM.jpg]
