# Supplementary material for: A novel molecular classification method for osteosarcoma based on tumor cell differentiation trajectories
Source: Bone Res. 2023 Jan 2;11:1. doi: 10.1038/s41413-022-00233-w (PMC9806110; doi:10.1038/s41413-022-00233-w)
Supplement: Supplementary file 34 — Supplementary Data [file 41413_2022_233_MOESM34_ESM.docx]

**Supplementary Data**

**Supplementary** **Fig. 1** Validation cohorts of the single-cell atlas of OS and CB samples. **a** Split UMAP plots of cells from each sample. **b** UMAP plots of OS and CB samples, color-coded for expression of the indicated marker genes. **c** Differences in the cell proportions of the 6 cell lineages between OS and CB samples. **d** Proportions of all cell clusters in each sample. **e** Grouping of cell clusters by similarity. Heatmap showing Pearson correlation coefficients between log-averaged expression profiles of clusters. **f** Distribution of unique molecular identifiers in each cluster.

**Supplementary** **Fig. 2** Differences in gene expression between different differentiation branches. **a** RNA velocity of mesenchymal cells. **b** Monocle2 method of the differentiation trajectory of OS cells. **c** Loess regression-smoothened gene expression of the indicated genes in pseudotime in the three differentiation trajectories. **d** Heatmap showing differences in GO pathway enrichment between the seven OS clusters. **e** Heatmap of expression levels of genes related to targeted therapies in OS-A2/B2/C2 clusters.

**Supplementary** **Fig. 3** Gene expression features and transcriptional programs of normal mesenchymal cells. **a** Differences in gene expression between BMSCs and adipocyte clusters. **b** Differences in gene expression between BMSCs and chondrocyte clusters. **c** Differences in gene expression between BMSCs and osteoblast clusters. **d** Heatmap showing differences in transcription factor activities between BMSCs, osteoblasts, chondrocytes, and adipocytes. **e** Differences in TF activity between BMSCs, osteoblasts, chondrocytes, and adipocytes. The top 8 activated TFs are marked in each cluster.

**Supplementary Fig. 4** Transcriptional program features of CSCs. **a** Heatmap showing differences in TF activity between the four subclusters of CSC-like cells. **b** Violin plots showing activity (left) and expression (right) of the four most activated TFs of the CSC cluster in 14 normal mesenchymal and OS cell clusters.

**Supplementary** **Fig. 5** Subclassing and distribution changes of lymphocytes and endovascular cells in OS and CB samples. **a** UMAP plots of T and B cells color-coded for identified clusters (left) or samples (right). **b** UMAP plots of endovascular cells color-coded for identified clusters (left) or samples (right). **c** Dot-plot heatmap of the most significant genes in the five T-cell clusters (top), the three B-cell clusters (middle), or the two endovascular clusters (bottom). **d** Differences in cell proportion between OS and CB samples in T-cell clusters (up), B-cell clusters (middle), or endovascular clusters (down). **e** Violin plots showing the expression of the specific subgroup markers in T cells (right), B cells (middle), or endovascular cells (left). * p < 0.05.

**Supplementary** **Fig. 6** Intercellular interactions in OS and CB samples. **a** Cell‒cell interaction networks estimated for CB (up, line width indicates the number of ligand‒receptor interactions, range: 19-231) and OS (down, line width range: 17-728) samples. **b** Significant ligand‒receptor interactions between OS-A2/B2/C2 and endothelial, osteoclast, or TAM clusters.

**Supplementary Table 1** Differentially expressed genes (DEGs) in 29 clusters

**Supplementary Table 2** Differentially expressed genes (DEGs) in mesenchymal cell clusters

**Supplementary Table 3** List of differentially expressed genes (DEGs) in CSC-like cells and BMSCs

**Supplementary Table 4** List of differentially expressed genes in CSCs vs. osteoblasts

**Supplementary Table 5** List of differentially expressed genes (DEGs) in CSC-like clusters and OS clusters

**Supplementary Table 6** List of differentially expressed genes (DEGs) between CSC-like cells and osteoblasts

**Supplementary Table 7** List of differentially expressed genes in BMSCs vs. adipocytes

**Supplementary Table 8** List of differentially expressed genes in BMSCs vs. chondrocytes

**Supplementary Table 9** List of differentially expressed genes in BMSCs vs. osteoblasts

**Supplementary Table 10** List of DEGs in OS-A2 vs. OS-B2

**Supplementary Table 11** List of DEGs in OS-A2 vs. OS-C2

**Supplementary Table 12** List of DEGs in OS-B2 vs. OS-C2

**Supplementary Table 13** Different pathway list of TARGET samples by the three groups

**Supplementary Table 14** Comparisons of clinical characteristics among the three groups of OS patients

**Supplementary Table 15** List of DEGs in CSC-like clusters

**Supplementary Table 16** List of DEGs in CSC vs. CSCL1 + CSCL2 + CSCL3

**Supplementary Table 17** List of differentially expressed genes in CSCL1 vs. CSCL2

**Supplementary Table 18** List of differentially expressed genes in CSCL1 vs. CSCL3

**Supplementary Table 19** List of differentially expressed genes in CSCL2 vs. CSCL3

**Supplementary Table 20** Different pathway list of the four clusters in CSC-like cells

**Supplementary Table 21** Different gene lists of myeloid cell clusters

**Supplementary Table 22** Different pathway list of myeloid clusters

**Supplementary Table 23** List of differentially expressed T-cell clusters

**Supplementary Table 24** Different gene lists of B-cell clusters

**Supplementary Table 25** List of differentially expressed genes in endovascular clusters

**Supplementary Table 26** Clinical characteristics of nine human cancellous bone samples

**Supplementary Table 27** Clinical characteristics of six osteoblastic OS samples
